# Sensory Cell Proliferation within the Olfactory Epithelium of Developing Adult *Manduca sexta* (Lepidoptera)

**DOI:** 10.1371/journal.pone.0000215

**Published:** 2007-02-14

**Authors:** Marie-dominique Franco, Jonathan Bohbot, Kenny Fernandez, Jayd Hanna, James Poppy, Richard Vogt

**Affiliations:** Department of Biological Sciences, University of South Carolina, Columbia, South Carolina, United States of America; University of Arizona, United States of America

## Abstract

**Background:**

Insects detect a multitude of odors using a broad array of phenotypically distinct olfactory organs referred to as olfactory sensilla. Each sensillum contains one to several sensory neurons and at least three support cells; these cells arise from mitotic activities from one or a small group of defined precursor cells. Sensilla phenotypes are defined by distinct morphologies, and specificities to specific odors; these are the consequence of developmental programs expressed by associated neurons and support cells, and by selection and expression of subpopulations of olfactory genes encoding such proteins as odor receptors, odorant binding proteins, and odor degrading enzymes.

**Methodology/Principal Findings:**

We are investigating development of the olfactory epithelium of adult *M. sexta*, identifying events which might establish sensilla phenotypes. In the present study, antennal tissue was examined during the first three days of an 18 day development, a period when sensory mitotic activity was previously reported to occur. Each antenna develops as a cylinder with an outward facing sensory epithelium divided into approximately 80 repeat units or annuli. Mitotic proliferation of sensory cells initiated about 20–24 hrs after pupation (a.p.), in pre-existing zones of high density cells lining the proximal and distal borders of each annulus. These high density zones were observed as early as two hr. a.p., and expanded with mitotic activity to fill the mid-annular regions by about 72 hrs a.p. Mitotic activity initiated at a low rate, increasing dramatically after 40–48 hrs a.p.; this activity was enhanced by ecdysteroids, but did not occur in animals entering pupal diapause (which is also ecdysteroid sensitive).

**Conclusions/Significance:**

Sensory proliferation initiates in narrow zones along the proximal and distal borders of each annulus; these zones rapidly expand to fill the mid-annular regions. These zones exist prior to any mitotic activity as regions of high density cells which form either at or prior to pupation. Mitotic sensitivity to ecdysteroids may be a regulatory mechanism coordinating olfactory development with the developmental choice of diapause entry.

## Introduction

**Figure 1:**
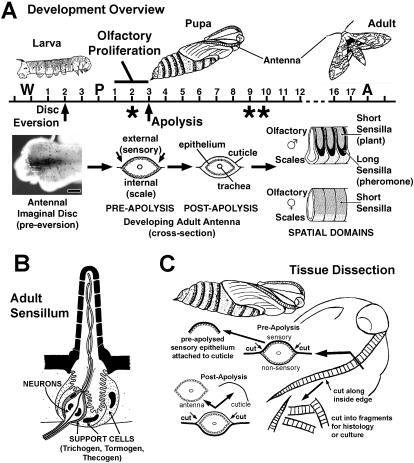
Olfactory Development, Sensillum Anatomy and Tissue Dissection. 1A. Overview of animal and antennal development in *M. sexta*. Time line represents days of development. W = onset of wandering behavior; P = pupal ecdysis; A = adult ecdysis. Asterisk under Pupa Day 2 marks time of developmental arrest for animals entering diapause. Asterisks under Pupa Days 9,10 mark times of specific apoptotic activity in olfactory epithelium (death of “t2 cells”; ref 6). See text for other details. 1B. Schematic of an olfactory sensillum. Olfactory neurons associate with three support cells that serve several functions including formation of the sensillum cuticle early in development and the secretion of perireceptor proteins (e.g. OBPs, ODEs) late in development. Distinct sensillum types differ in cuticle morphology, number of neurons and the branched state of the neuronal dendrites, as well as the combinatorial expression of sets of olfactory genes [ref 20], [64], [65]. 1C. Tissue dissection. A scalpel incision was made through the pupal cuticle along the inside perimeter of the developing antenna. For pre-apolysis animals, the underlying presumptive sensory epithelium was attached to the cuticle; cuticle with epithelium attached was processed for histological and tissue culture studies. For post-apolysis animals, removal of the pupal cuticle allows access and removal of the developing antennal tissue within.

The adult antennae of *Manduca sexta* are highly patterned appendages deriving from imaginal primordia that initiate growth during the final (5th) larval instar, and continue to develop throughout the metamorphic pupal stage. Larvae also possess antennae; a pair of fifth instar larval antennae of *M. sexta* possess around 20 olfactory receptor neurons each, distributed among 6 olfactory sensilla [1]–[4]. During metamorphosis, the larval antennae are replaced by a pair of structurally complex adult antennae, each with about 250,000 olfactory receptor neurons distributed among some 100,000 olfactory sensilla [5]–[8]. Each adult antenna, male and female, is divided into about 80 nearly identical segment-like annuli; and each annulus is divided into a sensory region containing the olfactory sensilla and a largely non-sensory region containing scales and a very small number of sensory structures **(** **Fig. 1a** **)**. In male antennae, each sensory region is further divided into a peripheral domain containing the sex-pheromone specific sensilla trichodea surrounding a central domain containing several classes of intermixed sensilla responsive to plant volatiles, including short sensilla trichodea, two morphologically distinct classes of sensilla basiconica as well as sensilla coeloconica [8]. The numbers of sensilla in the pheromone and plant-volatile sensitive domains are equivalent. The sensory region of each female annulus is more homogeneous, appearing to have only a single domain of intermingling sensilla types but lacking the pheromone-specific long sensilla trichodea, though the distributions of specific sensilla types do differ across the epithelium [9], [10]. Emerging data support views that distinct classes of sensilla process different classes of odorant [9]–[15]. This study identifies early temporal/spatial patterns in adult antennal development that may contribute to the establishment of adult spatial patterns and sensilla phenotypes.

Each adult antenna derives *de novo* from an imaginal primordium which grows inward from a ring of cells surrounding the base of the larval antenna; the majority of disc growth occurs during the final larval instar **(** [5], [16]–[18]; Fernandez and Vogt, unpublished) **(** **Fig. 1A** **)**. Larvae undergo an abrupt change from a feeding state to a wandering prepupal state 4–5 days before pupation. About mid-way between this change and actual pupation, the antennal imaginal primordia evert outward from the animal's body forming tubular structures consisting of a monolayer epithelium. Antennal eversion coincides with head apolysis, or the detachment of the head and antennal epithelium from the overlying larval cuticle in anticipation of molting. Apolysis creates a fluid filled external chamber into which the antennal primordia evert. The larval antenna epithelium remains connected to the larval antennal cuticle through this process, and the separation of larval head epithelium and cuticle may contribute to eversion through this sustained connection (Fernandez and Vogt, unpublished). The elongated antennal primordia have a folded or corrugated morphology; the concave valleys of each fold correspond to the annular boundaries of the adult antenna. During the actual pupation event, or shedding of larval cuticle, the elongated and everted primordia are stretched down along the outer body surface, initially pulled down by the shedding larval cuticle through the retained loose connection between the distal tip of the disc (the old larval antenna epithelium) and larval antenna cuticle, and further stretched by hydrostatic pressure of circulating hemolymph. At the end of this process the developing adult antennae lie on the outer surface of the pupa with the presumptive sensory neuron epithelium facing outward and the scale producing epithelium facing inward. The antennal epithelium secretes pupal cuticle beginning about 24 hrs before pupation and continuing until at least 24 hrs after pupation (a.p.). During the first three days a.p., the antennal epithelium remains attached to its pupal cuticle. However, about 72 hrs a.p. the antennal epithelium undergoes apolysis, detaching from the pupal cuticle; following this apolysis the antenna is physically unconstrained to undergo the morphogenesis required to form the adult antenna, and proceeds with secretion of the adult cuticle and the formation of adult olfactory sensilla.

Each olfactory sensillum has a cellular component consisting of one to several chemosensory neurons plus three support cells (trichogen, tormogen, thecogen) [19]–[21] (**Fig. 1B** **)**. In general, this collection of cells derives from the proliferation of sensory precursor cells originating within the presumptive olfactory epithelium. In Lepidoptera, the cells comprising individual sensilla have been suggested to be clonally related, originating through mitotic divisions from a single sensory mother cell [6], [22]–[25]. However, in *Drosophila*, the cells comprising individual olfactory sensilla may be somewhat less related, originating through an initial and coordinated association of neighboring cells which subsequently proliferate to yield the neurons and support cells [26]–[29].

In *M. sexta*, Sanes and Hildebrand [5], [6] employed light and electron microscopy to study the development of the male antenna and the male specific pheromone sensitive trichoid sensilla. As part of their study, the developmental timing of mitotic activity (S-phase) producing olfactory sensory cells was characterized by chromosomal incorporation of tritiated thymidine [6]. Animals were injected at various times from pupation up to 80 hrs a.p., and examined around 9–10 days a.p. when cellular phenotypes could be recognized. Sensory cells could only be labeled by injections made between 25 and 60 hrs a.p., suggesting that, in male *M. sexta*, all sensory proliferative events giving rise to adult olfactory sensilla were restricted to this time period, prior to apolysis during the period when the epithelium is still attached to the cuticle.

The diversity of identifiable cellular phenotypes in the developing adult *M. sexta* antenna make this tissue an attractive comparative system in which to test specific cellular and molecular models applicable to olfactory metamorphosis. In the current work, a variety of histological approaches were employed to reconfirm and refine observations of some of the early developmental events, and especially the proliferative events, originally observed by Sanes and Hildebrand [6]. These proliferative events are described in both normal developing animals and animals entering and leaving diapause (winter dormancy). Evidence is presented that the ecdysteroid hormones are involved in the regulation of these proliferative events. It is suggested that the temporal pattern of proliferation and its ecdysteroid sensitivity accommodates the developmentally plastic decision of continuous development during a growing season vs. diapause based dormancy during an over wintering period. This study provides a baseline for continuing studies characterizing the roles of ecdysteroids and regulatory genes in determining cellular phenotypes in the *M. sexta* olfactory system.

## Materials and Methods

### Animals and Tissues

*Manduca sexta* were obtained as fertilized eggs from Dr. L. M. Riddiford (University of Washington, Seattle), and reared at 27°C on a 16∶8 L∶D cycle. For the majority of studies, pupae were aged from the time of pupation (hours after pupation: “hrs a.p.”) using time-lapse and infrared video recording to identify the exact time when individual animals shed their cuticle (t = 0), an activity that occupies about 5 minutes. Inactive 5^th^ instar larvae, late in their wandering phase but at least 1 day prior to pupation, were placed in clear glass shell vials (1″ diameter) to allow camera visualization; vials and camera were situated within our normal growth chamber. For the initial BrdU studies, pupae were developmentally staged using anatomical criteria of Oland *et al.* [30]: pupal Stages 1 to 4 were identified by progressive changes in body color and by the visual appearance of the leg trachea; we have equated these stages with approximate hrs a.p. in figures where this data is presented. For diapause studies, pupae entering or in diapause were obtained from Dr. Riddiford's culture facility, derived from larvae cultured at 27°C on a short day length light cycle (12∶12 L∶D). Animals destined to diapause pupate normally and develop for approximately 2 additional days before becoming dormant; animals studied entering diapause were aged from the time of pupation by video recording. Animals in diapause, used to study events associated with reinitiation of development, were taken from a population which had been held 48–52 days in diapause under the short day length rearing conditions described above.

It was convenient to organize our study around antennal apolysis, which occurred at around 72 hrs a.p. in non-diapausing animals. The anatomical staging criterion Stage 3 occurred prior to apolysis in our study, differing from Stage 3 in other studies, such as that of Rössler *et al.*, [31], in which Stage 3 occurred as much as 24 hrs after antennal apolysis (Lynn Oland, personal communication). Such difference may be due to our lack of familiarity with the anatomical staging criteria, from genetic differences between two laboratory strains, or from differences in rearing conditions, but are important to note for data comparison.

The isolation of tissue characterized in this study is illustrated in **Fig. 1C**. To isolate pre-apolysis sensory epithelium from pupae (pupation through about 72 hrs a.p.), the outward facing antennal pupal cuticle with epithelium attached was removed using a scalpel and immediately fixed in 4% paraformaldehyde (PFA) in PBS-Tx(0.3%) (10 mM Na phosphate, 150 mM NaCl, pH 7.0, containing Triton X at 0.3%) and processed as described below. To isolate post-apolysis tissue (older than 72 hrs a.p.), the entire pupal antennal cuticular chamber was first removed, pinned outside-down under saline, the inward facing cuticle removed and the antenna floated free. This post-apolysis tissue was pinned outstretched under fixative (4% PFA in PBS-Tx(0.3%)), and openings were made using a scalpel to allow chemical access to the interior cells. For all dissections (and injections), animals were first anesthetized on ice for at least 15 minutes. All dissections used miniature scalpel blades manually formed from single edge razor blades using a blade breaker (George Tiemann & Co., Hauppauge, New York #160-346).

### Detecting mitotic activity and DNA synthesis: BrdU and H3 phospho-Histone

#### BrdU labeling

Accumulated mitotic activity (S-phase) was visualized using 5-Bromo-2′-deoxy-uridine (BrdU) Labeling and Detection Kit II (Roche). Pre- and post-apolysis pupae were injected into the head near the base of the antennae with 1 ul per gram body weight of 10 mM BrdU, and allowed to continue developing 12–15 hours at 27°C, 16∶8 L∶D cycle. Following this period, tissue was dissected, fixed in 4% PFA in PBS-Tx(0.3%) at 4°C, and permeabilized in 2 N HCl, PBS-Tx(0.3%) at 4°C for 30 min. Tissue was blocked (1% Bovine Serum Albumin (BSA), 1% lysine, 1% glycine in PBS-Tx(0.3%); 4°C, 1 hr), incubated in anti-BrdU monoclonal antibody (supplied with labeling kit, 1∶10 dilution in blocking solution; 4°C, overnight). Anti-BrdU was visualized using anti-mouse IgG-alkaline phosphatase (Fab fragment supplied with Labeling and Detection Kit and packaged with nuclease activity, 1∶10 dilution in PBS-Tx(0.3%); 4°C, overnight). Alkaline phosphatase activity was detected with nitroblue tetrazolium (NBT) and 5-bromo-4-chloro-3-indolyl phosphate (BCIP) at 25°C using recommended procedures, and stopped in cold 10 mM TE-Tx(0.1%) (10 mM Tris-HCl, 1 mM EDTA, pH 7.5, 0.1% Triton-X 100).

#### H3 labeling

Instantaneous mitotic activity (M-phase) was visualized using antiserum which recognized the phosphorylated state of the H3-histone [32]. Tissue was dissected, fixed, and permeabilized as described above for BrdU. Tissue was blocked (PBS, 1% BSA, 1% Non-Fat Dry Milk, 1% TritonX, 5% normal goat serum (NGS), 0.05% NaAzide; 4°C, overnight), and then incubated with Phospho Histone H3 Mitotic Marker antibody (Upstate Biotechnology, #06-570; 1∶1000 dilution; 2 days at 4°C) in the same blocking solution. H3 antibody was visualized using goat anti-rabbit IgG-alkaline phosphatase (Sigma A-3937, 1∶1000 in PBS with 1% Triton X); alkaline phosphatase was detected as described above for BrdU. Alternatively, H3 antibody was visualized using Cy2 labeled secondary antibody (Jackson Immuno Research Laboratories, Inc., Cy2-conjugated AffiniPure Goat Anti-Rabbit IgG (H+L), 1∶200 in PBS-Tx), applied under identical conditions as described above, and visualized by Multi-photon Laser Scanning Microscopy (MPLSM) (890ex/525em nm; ex = excitation, em = emission; Cy2 was subject to rapid bleaching under these conditions (see “Microscopy and Image Processing” below).

### Propidium Iodide (PI) labeling of nuclei

To visualize nuclei, tissue was dissected, fixed in 4% PFA in PBS-Tx(0.3%), and treated in RNase A (0.2 mg/ml in PBS-Tx(0.3%), 30–60 minutes, room temperature) to reduce the cytoplasmic fluorescence due to interaction between RNA and propidium iodide (PI). Tissue was then incubated in PI (Sigma P4170, 20 ug/ml in PBS-Tx(0.3%) from 10 mg/ml stock in H_2_O, 30 minutes, room temperature), washed in PBS-Tx(0.3%), and stored in TE-Tx(0.1%) until visualization by MPLSM (770ex/605em nm) or confocal microscopy (568ex/598em nm). In some studies, PI was used as a counter stain; in these cases, following other treatment, tissue was treated with RNase A and PI as described above.

### Pupal Cuticle Protein (PCP) In situ hybridization

For whole mount *in situ*-hybridization, pre-apolysis tissue was dissected as described earlier, incubated 4% PFA in PBST (overnight, 4°C), dehydrated to 70% Methanol, and stored at −20°C. Antisense and sense RNA probes were synthesized from *M. sexta* PCP (accession # AY585211, ligated into pPCR-Script Amp SK(+) (Stratagene)) and amplified by PCR using M13 forward and reverse primers, after Vogt *et al.* [4]. For antisense or sense probes, PCR product was truncated using either BamH I or Not I restriction enzymes as appropriate, gel purified (GENECLEAN Turbo, Q-Biogene), and transcribed to RNA using either T3 or T7 RNA polymerase (Stratagene) in the presence of digoxigenin labeled UTP (Roche, after Rogers *et al.* [33]) and 40 units of RNaseOUT (Invitrogen). RNA probes were then alkaline-degraded to approximately 200 bases after Byrd *at el.*,[34]. *In situ* hybridizations were modified after Byrd *et al.* [34] and Rogers *et al.* [33]. Tissues were selected from 70% methanol storage, rehydrated to PBS-Tw(0.1%) (PBS containing Tween 20 at 0.1%), treated with Proteinase K (10 µg/mL; 20 min at 37°C), fixed in 4% PFA/0.5% glutaraldehyde in PBS-Tw(0.1%) (30 min, 20°C), prehybridized (0.6 M NaCl,; 10 mM Tris, pH 7.5; 2 mM EDTA, pH 8; 1X Denhardt's solution; 70 µg/mL herring sperm DNA; 100 µg/mL tRNA; 0.2% Tween 20; 68°C, overnight) and hybridized with approximately 100 ng/mL PCP probe (in prehybridization solution containing formamide at 50%; 68°C, 15 hrs). Tissues were washed in PBS-Tw(0.1%), blocked in PBS-Tx(1%) containing Blocking Reagent (1%; Roche) and normal donkey serum (5%) (2 hrs, 20°C), and incubated with anti-digoxigenin antibody (1/200; whole IgG, Roche) in PBS-Tx(1%) (overnight, 4°C). Following washes in PBS-Tx(0.3%), tissues were incubated with Alexa Fluor 488 donkey anti-sheep antibody (Molecular Probes) in PBS-Tx(1%) (overnight, 4°C). Following washes in PBS-Tx (0.3%), tissues were treated with RNase overnight and stained with PI as described elsewhere.

### Ecdysteroid Studies, Including Induced Development from Diapause

20-hydroxyecdysone (20HE, Sigma) was dissolved in 10% aqueous isopropanol (3 mM, 1.45 mg/ml); α-ecdysone (gift of R. Lafont via L. M. Riddiford; see Hiruma et al. [35]) was dissolved in absolute ethanol (4.3 mM, 2 mg/ml). Concentrations were determined spectrophotometrically (ε_240_ = 12,670, ε_242_ = 12,388 respectively). The α-ecdysone had been previously HPLC purified from other contaminating ecdysteroids [36]. The non-steroidal ecdysteroid analog RH5992 (gift of Rohm and Hass Company, Spring House, PA) was dissolved in 10% aqueous isopropanol (10 mg/ml). Aliquots of hormone and analog stocks were added directly to the culture medium to achieve desired concentrations.

Tissue was cultured in the absence or presence of hormone, following procedures modified from Riddiford *et al.* [37] and Vogt *et al*. [38]. Dissected tissue was rinsed and cut into segments in sterile Grace's Insect Medium (Gibco BRL, # 11590-056) and transferred into 24-well culture plates. Each well contained a small amount of sterile glass wool for tissue support and 0.75 ml of Grace's medium supplemented with BrdU (20 µM) with or without hormone or hormone agonist as indicated (note: glass wool can physically damage tissue, and has since been eliminated from this procedure). The culture plates were placed in closed plastic boxes infused with a slow delivery of 95%O_2_/5%CO_2_ gas at 27°C. The boxes were placed on an orbital shaker under gentle rotation, and tissue was incubated for indicated times. At the end of the incubation, tissue was processed to detect BrdU incorporation or phospho-H3 antigen as described above.

To induce development in diapausing pupae, 20 ug 20HE (100 ul) was injected into mid-lateral dorsal thorax (animal masses ranged 5.2–6 gm); 20HE was diluted with Grace's medium from 2 mg/ml stocks (10% isopropanol in H_2_O). Injection sites were surface cleaned with 70% ethanol, and injection holes were sealed with melted beeswax-rosin (1∶1 mixture).

### Microscopy and Image Processing

For light microscopy, tissue was photographed under dark field using color transparency film (subsequently digitally scanned) on either an Olympus BX60 compound or SZH10 stereo microscope. For Confocal and Multiphoton Laser Scanning Microscopy (MPLSM), tissue was imaged using a BioRad 1024ES(2P) system equipped with an argon/krypton laser and a Coherent Verdi/Mira Ti/Si laser (5 watt, with extended wavelength optic set) MPLSM employed a non-descanned external detector. All figures were processed using Adobe Photoshop.

## Results

### Examination of pre-apolysis mitotic activity based on BrdU incorporation

Mitotic activity (S-phase) and DNA replication were examined the presumptive sensory epithelium of developing male and female adult *M. sexta* antennae. BrdU incorporation was examined during the first three days following pupation, up to the time of apolysis. Apolysis is the detachment of the developing epidermis from the overlying pupal cuticle, and occurs about 72 hrs after pupation (a.p.). Although apolysis and mitotic activity appear to be independent events (discussed later in relation to diapause studies), apolysis is a convenient event around which to characterize early antennal development, and the mitotic activity giving rise to cells of olfactory sensilla was previously reported to have completed by this time [6].

**Figure 2:**
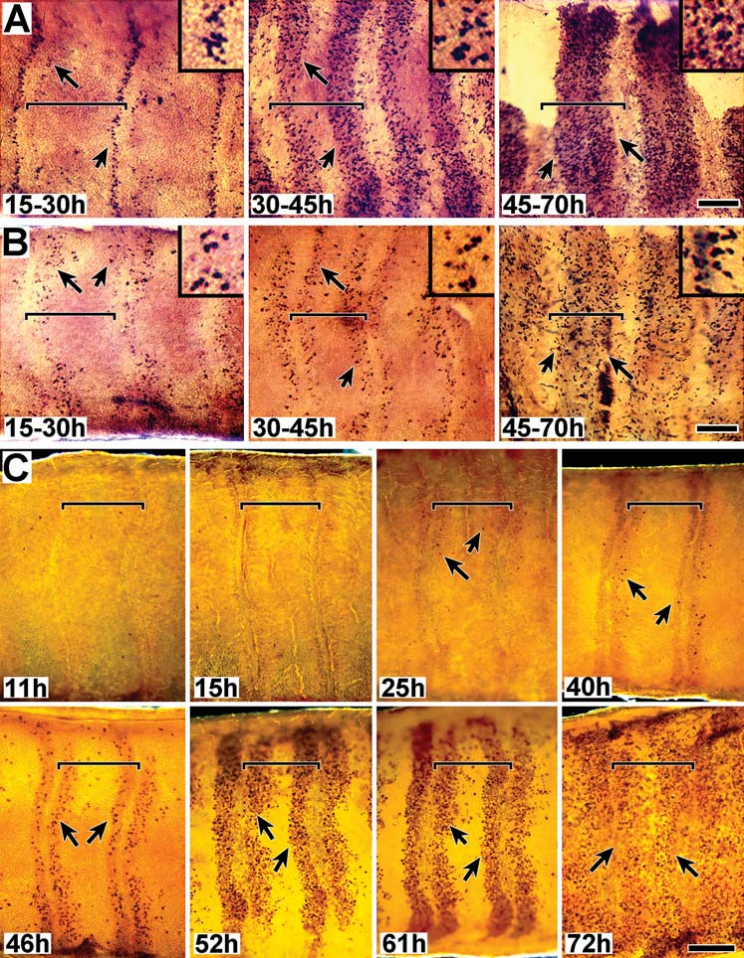
DNA replication in sensory epithelia. A,B. Mitotic activity studies using BrdU incorporation (male and female). Developing sensory epithelia of male (A) and female (B) antennae are shown in whole-mount, attached to cuticle. Animals were injected with BrdU and tissue was dissected 15 hrs later; approximate injection and dissection times after pupation (a.p.) are indicated, based on developmental stages (stages 1–3). The tissue shown in 2B (45–72 h) is undergoing apolysis; a portion of the epithelium was torn during its removal and is thus missing. DNA replication was visualized using phosphatase tagged BrdU antiserum. Arrows indicate representative BrdU staining. Size bar (B, 45–70 h): 150 u (A–F); 50 u (inserts). C. Mitotic activity studies using phospho H3 visualization (male). Developing sensory epithelia, all male, are shown in whole-mount, attached to cuticle. Animals were dissected at the indicated times a.p. Nuclei undergoing mitosis were visualized using phospho-H3 antiserum. Arrows point to representative positive staining; no detection was observed prior to 24 hrs. Vertical lines visible along annular borders, especially noticeable in the 11h and 15h tissues, are part of the underlying (outer) pupal cuticle; annular borders align with these cuticular structural features. Size bar (C, 72 h): 200 u. For all figures (A–C), distal is left, proximal is to the right. Sensory epithelium was examined still attached to the outward facing antennal pupal cuticle. Annular boundaries and the proximal-distal orientation of the tissue were easily identified by distinct markings in the overlying pupal cuticle. Tissues were photographed in darkfield. Horizontal brackets mark the limits of single annuli within each panel; proximal is to the left and distal to the right.

BrdU incorporation followed a temporal-spatial progression, initiating along the proximal and distal borders of each presumptive annulus and then expanding into the mid-annular region **(** **Fig. 2A,B** **)**. BrdU was injected into pupae (n>50 for each sex) at times ranging throughout the first 3 days a.p. (developmental stages 1–3); tissue was removed 12–15 hrs later and analyzed for evidence of incorporation into nuclei (i.e. chromosomal DNA). In males (A) and females (B), BrdU incorporation initiated in two narrow zones, one along the proximal and one along the distal border of each annulus. As development progressed, both zones broadened towards the mid-annular region, appearing to fill the entire annulus by apolysis. Figure 2A (15–30 h), male tissue, shows a higher level of mitotic activity in the proximal region of each annulus. However, Figure 2B (15–30 h), female tissue suggests the opposite, a higher level of mitotic activity in the distal region. These differences more likely reflect differences in timing of BrdU injection or developmental staging differences rather than sex; Figure 2C (25 h and 40 h), also male tissue, shows higher levels of mitotic activity in the distal region (see below). The female tissue (2B) also appears to be more sparsely labeled than male tissue (2A); these differences are most likely due to differences in BrdU incubation times, as males and females are known to contain similar numbers of sensory cells [9], [10].

These BrdU studies suggest that the mitotic activity giving rise to cells comprising olfactory sensilla, both trichoid and basiconic, initiates in narrow zones along the proximal and distal annular boarders and spatially expands into the mid-annular region by the time of apolysis, and that a similar pattern is followed for both male and female tissue.

### Examination of pre-apolysis mitotic activity based on H3 histone phosphorylation

DNA replication studies based on incorporation of BrdU or tritiated thymidine present blurred pictures of the temporal-spatial patterns of mitotic activity because an extended period of time intervenes between application and visualization of the probe. Immunodetection of the phosphorylated state of the H3 histone, after Wei et al. [32], provides a more instantaneous view of mitotic activity (M-phase). Phospho-H3 antiserum was therefore used to obtain a higher resolution view of the temporal and spatial patterns of mitotic activity during the pre-apolysis period.

Pre-apolysis mitotic activity occurs in three phases of temporal activity. **Fig. 2C** shows a representative timed series of tissue aged from 11 hrs a.p. to 72 hrs a.p. All tissues were taken from the same rearing group of animals (i.e. identical growth conditions) and were processed together and stained for identical times. The general and consistent pattern observed was (phase 1) no detectable mitotic activity occurring during the first 24 hour period a.p., (phase 2) a low level of mitotic activity occurring during the second 24 hr period, and (phase 3) a high level of mitotic activity occurring during the third 24 hr period. Spatially, M-phase cells initially appeared in narrow zones along the proximal and distal annular borders. These zones became broader with time, appearing to expand towards the mid-annular region (as observed in the BrdU studies).

**Figure 3:**
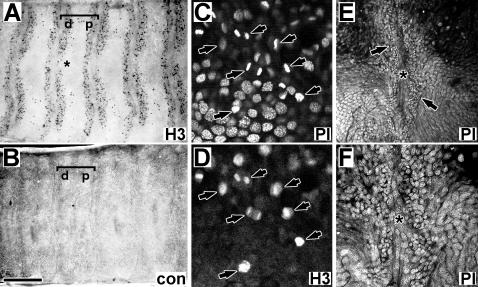
Mitotic activity and early appearance of high density zones (male). A,B. Mitotic activity at 52 hrs a.p. “A” shows cells visualized with anti-phospho H3 antiserum; “B” is tissue visualized using control serum (anti-*Apol*SNMP1, 1∶1000 dilution, after Rogers et al., 1997). Brackets mark single annuli; “d” and “p” mark distal and proximal zones. Asterisk marks discontinuous region in distal zone (see text). C,D. Mitotic figures. Tissue (67.5 hr a.p. male) was double stained with PI and anti-phospho H3 antibody (visualized with Cy2 labeled secondary antibody). Arrows point to identical cells in “C” and “D”. S E,F. High density clusters of cells along annular border as detectable by 2 hrs a.p. Images show male sensory epithelium (2 hr a.p.) centered on an annular boundary (asterisks), with high density zones evident on either side of this boundary (brackets). “E” and “F” are the same tissue at different magnification; asterisks mark identical positions as well as the annular boundary. Size bar: 350 u (A,B); 24 u (C,D).90 u (E); 45 u (F);

The mitotically active zones illustrated in **Fig. 2C** were restricted to the presumptive sensory epithelium, underlying the outward facing portion of the antennal pupal cuticle, and did not extend into the scale forming regions of the developing antenna. The lateral termination of these zones is visible in several images, including the 46 hr (upper edge) and 52 hr (lower edge) tissues in **Fig. 2C**, as well as in **Fig. 3A** (lower edge). The proximal and distal zones of each annulus initially differed consistently in shape and size, with the distal zone starting out broader in the proximal-distal dimension and shorter in the lateral dimension than the proximal zone. The distal zone was also often observed as discontinuous along the mid-line of the antenna during the early phase of mitotic expansion (**asterisk,** **Fig. 3A**).

### Direct examination of nuclei and nuclear density changes in pre-apolysis tissue by chromosomal fluorescence (propidium iodide) and MPLSM

Cell division results in the immediate partitioning of a cell into two smaller cells which may later enlarge and change shape; indirect evidence of recent mitotic activity may therefore be the presence of small cells among a field of larger cells. Such regions of small vs. large cells can be distinguished as regions of high density vs. low density nuclei. Regions of high and low density nuclei within the developing epithelium were therefore directly observed by staining tissue with propidium iodide (PI, following RNase treatment) and visualized using multiphoton laser scanning microscopy (MPLSM).

**Figure 4:**
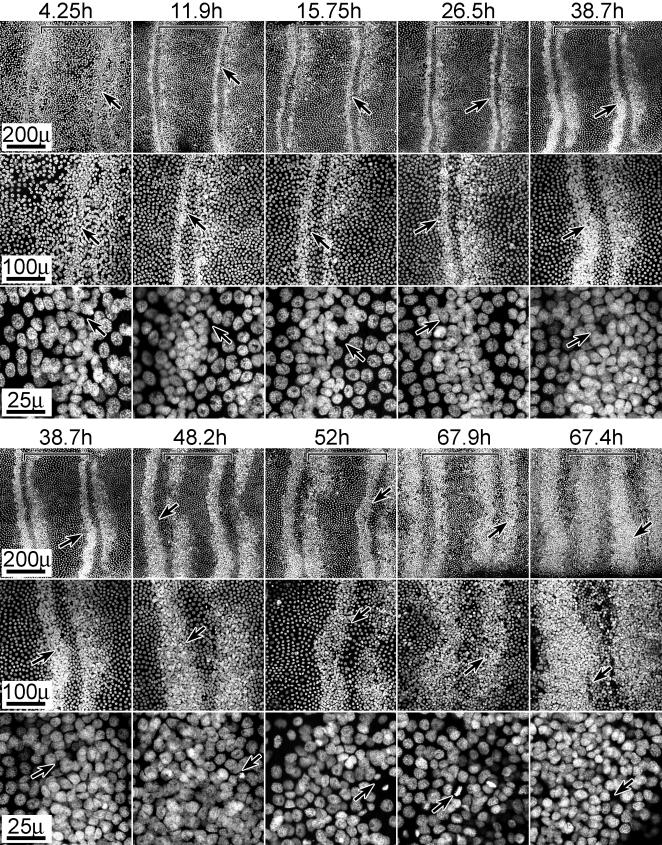
Nuclear stain (PI) reveals zones of high density nuclei which expand with age. Tissues (male) of indicated ages (hours a.p.) are shown stained with PI and at three magnifications. Arrows mark identical positions at each magnification; brackets indicate the boundaries of a single annulus (distal is left). Arrows in high magnification panels (25 u) of 52 hr and 67.9 hr tissues additionally point to mitotic figures. The 67.9 and 67.4 h a.p. tissues are shown in sequence of developmental progression rather than temporal progression; the differential degree of progression in these tissues is presumably due to temporal developmental asymmetry between individual animals. For all images, distal is left, proximal is to the right. Size bars are shown for each row.

**Figure 5:**
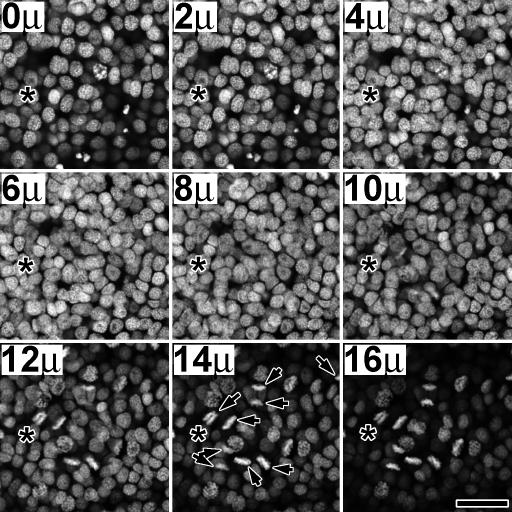
Z-series through a high density zone. Tissue (55.8 hr a.p. male) was stained with PI and imaged at 2 u increments from hemolymph (0 u) to cuticle (16 u) (MPLSM). Mitotic figures appeared to be restricted to the cell layer adjacent to the cuticle. Asterisks mark identical positions in each frame. Arrows in the 14 u panel indicate mitotic figures, which can also be seen from panel 10 u–16 u, indicating that mitotic activity is restricted to an epithelial layer near the cuticle. Lens: Nikon Plan Fluor 40x/0.75 dry. Size bar: 25 u.

The spatial pattern of high v. low density nuclei is shown in tissues aged from 4.25 hrs to 67.9 hrs a.p. (**Fig. 4**). The 38–67 hr a.p. images show zones of high density nuclei lining the proximal and distal annular borders; these zones expand towards the mid-annular regions as development progresses. This expansion parallels the expansion of mitotic zones already described **(** **Fig. 2** **)**. Mitotic figures (separated chromosomes during telophase) were observed within these high density zones in tissues aged older than 24 hr a.p., confirmed by double labeling with Propidium Iodide and phospho H3 antibody (**Fig. 3C,D**). Z-series analysis through the high density regions revealed several layers of nuclei (3–4), with mitotic nuclei always in the layer nearest the cuticle **(** **Fig. 5** **)**. We presume, therefore, that the expansion of these high density nuclear zones is due to increased and expanding regions of mitotic activity.

**Figure 6:**
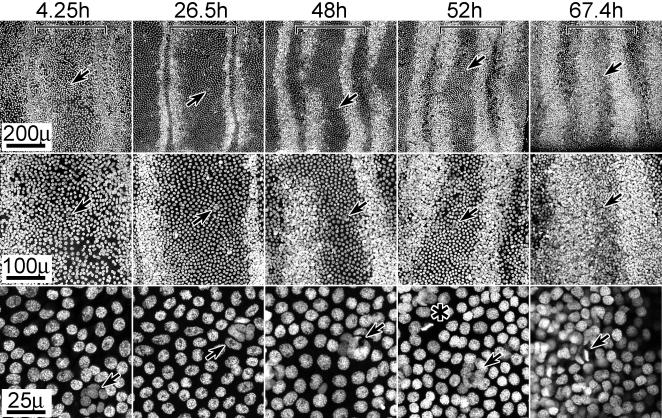
Nuclear stain (PI) reveals isolated clusters of nuclei in mid-annular regions, already present at pupation. Select tissues from the series shown in Fig. 4 are shown, focusing on mid-annular regions. Arrows mark identical positions at each magnification; brackets indicate the boundaries of a single annulus (distal is left). Arrows in high magnification panels (25 u) (4.25–52 hrs a.p.) additionally point to clusters of high density nuclei within the mid-annular regions; the high density zone has expanded into this region in the 67.4 hr tissue, obscuring the observation of these clusters. Note mitotic figures in the high magnification (25 u) 52 hr (asterisk) and 67.4 hr (arrow) panels. Size bars are shown for each row.

The 4–38 hr a.p. images **(** **Fig. 4** **)** reveal that the high density nuclear zones are present prior to any observed mitotic activity. Again, high density zones were consistently observed lining the proximal and distal annular borders at the earliest time points. These zones appear less organized at 4.25 hr than at 11.9 hr a.p., but once stabilized, the width of these zones remained constant to about 24 hrs a.p. after which they began to expand in a pattern that paralleled the expansion of mitotic zones (e.g. **Fig. 2**). The high density zones were visible as early as 2 hrs a.p. (**Fig. 3E,F**) suggesting they form earlier and possibly prior to actual pupation. We do not know whether these initial zones result from reorganization of cells or from mitotic activity. When animals were injected with BrdU as early as 12 hr prior to pupation and dissected 12 hr after pupation, no BrdU incorporation was observed in this tissue (data not shown); thus, if these zones arose from mitotic activity, that activity would have occurred earlier than 12 hr before pupation.

Focusing attention on the mid-annular regions, small clusters of cells were already apparent in the 4.25 hr a.p. tissue (**Fig. 6**). These cells are reminiscent of sensilla cell clusters already having undergone mitosis [25], suggesting that if these do indeed represent a specific class of sensillum, this class presumably undergoes its proliferative activity much earlier, presumably prior to 12 hrs. before pupation. These mid-annular cell clusters might represent coeloconic sensilla which are present in adult antenna in similar numbers and distribution [8]. In *Drosophila* antennae, precursor cells of coeloconic sensilla are known to appear prior to those of trichoid and basiconic sensilla [28], [29].

**Figure 7:**
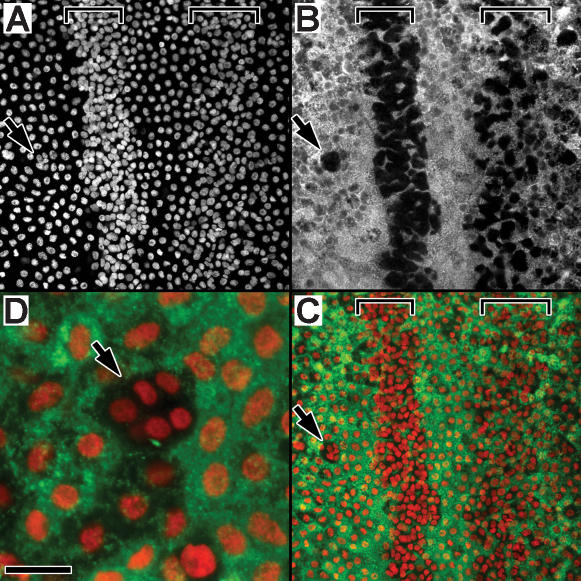
High density cells do not express Pupal Cuticle Protein (PCP). *In situ* hybridization of tissue (48 hrs a.p., male) using antisense RNA PCP probe (green, cytoplasm), counterstained with PI (red, nuclei). Brackets mark high density nuclear zones; arrows point to a cluster of high density nuclei within the mid-annular region of low density nuclei. “A”, PI stain (white) of nuclei, showing proximal (p) and distal (d) high density zones of two adjacent annuli separated by an inter-annular region. “B”, the same tissue, visualizing PCP expression (white). “C”, color composite showing nuclei (red) and PCP expression (green); black unstained regions correspond to high density zones. “D”, a high density mid-annular nuclear cluster (arrow, similar to the cluster indicated in A–C) within the low density mid-annular region; nuclei are shown in red (PI) and PCP expression in green. PCP expression (green) is restricted to the low density cells, but absent from the cells associating with high density nuclei. PCP data is from a larger study of PCP expression in *M. sexta* (Bohbot and Vogt, in preparation). Size bar: 50 u (A–C), 12.5 u (D).

High and low density cells have different functions, suggested by the differential expression of a cuticle gene referred to as PCP (Pupal Cuticle Protein); high density cells do not express this cuticle gene **(** **Fig. 7**). During the first few days of the pupal stage, the presumptive sensory epithelium of the antenna is simultaneously performing two major tasks, producing pupal cuticle and producing the cells which will comprise the olfactory sensilla. PCP expression is evident throughout the epithelium, except in the cells which comprise the high density zones **(** **Fig. 7A–C**) and the small mid-annular clusters **(** **Fig. 7D**). The differential expression of PCP within the epithelium, and notably not in the high density cells is additional evidence that these high density cells have a specific fate within the antenna, a fate we are suggesting is to develop into olfactory sensilla. Close examination reveals that PCP expressing cells do in fact occur within the high density zone, but only in large cells which intermingle with the smaller high density sensory precursor cells (visible in **Fig. 7B,C**). Z-series analysis (not shown) indicates that the nuclei of the PCP expressing cells within these zones reside near the basal region of this epithelium, to the hemolymph side of the high density nuclei, with PCP-positive cytoplasm extending through the epithelium from hemolymph side to cuticle side.

### Sensory Proliferation in Animals Entering and Exiting Diapause

The temporal pattern of mitotic activity suggests that, while some activity does initiate around 24 hrs a.p., there is a significant increase in activity beginning around 48 hrs a.p. Forty eight hours a.p. is a notable time point because it is at this time that *M. sexta* growing under short day light conditions would enter diapause, a state of developmental arrest during which an insect survives a period of seasonal environmental extreme [39]. *M. sexta* diapause as pupae, induced by decreasing day length experienced as larvae. Diapausing pupae develop for about 2 days and then arrest because the brain neuropeptide prothoracicotropic hormone (PTTH) is not released [40]–[42]; PTTH stimulates ecdysteroid synthesis and release, and its production is regulated by environmental factors such as day length. Animals will reinitiate development in the spring in response to environmental stimuli such as increased temperature [43], stimulated by PTTH release and the resulting production of ecdysteroids. The coincidence of the time of developmental arrest in diapausing animals with the observed increase in antennal mitotic activity around 48 hrs a.p. in non-diapausing animals prompted us to analyze sensory proliferation in animals entering and exiting diapause.

Diapause can be experimentally induced and sustained for several months by raising animals on a short day cycle (12∶12 light:dark) [44]. Diapause can be experimentally broken by injecting pupae with 20HE [45]. Hormone injection is thought to trigger the production and release of endogenous ecdysteroids [39].

**Figure 8:**
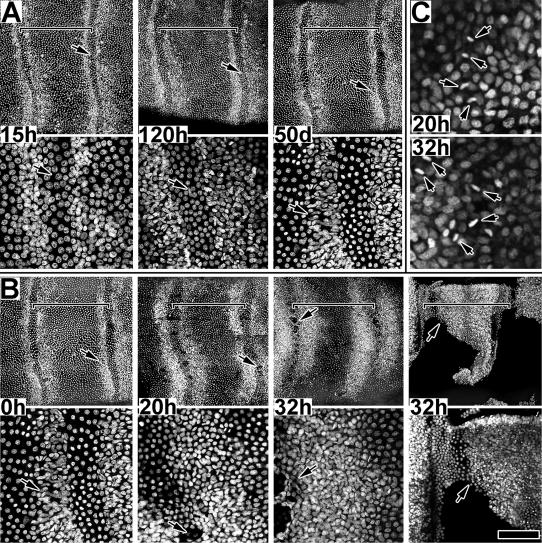
Spatial dynamics of high density zones entering and exiting diapause. A. Presumptive sensory epithelium from animals (males); 15 hr a.p. tissue if from normal developing animal while 120 hr and 50 day a.p. tissue is from animals in diapause. Two magnifications are shown for each tissue; arrows mark identical positions for each pair. Tissues are aged in hours (h) or days (d) a.p. Size bar (15 h–50 d): 200 u (upper), 50 u (lower). B. Presumptive sensory epithelium from animals exiting diapause, stimulated by injection of 20E at t0 (0 h). Two magnifications are shown for each tissue; arrows mark identical positions for each pair. Tissues are aged in hours (h) following hormone injection. Tissue from two animals, both aged 32h after hormone injection, are shown to note the temporal variation in response to the treatment. Size bar (0 h–32 h): 200 u (upper), 50 u (lower); Size bar (32 h*): 200 u (upper), 100 u (lower). C. Tissue exiting diapause, 20 and 32 h following hormone injection; arrows indicate mitotic figures within a high density zone. Size bar: 25 u (upper), 50 u (lower). For all figures, brackets mark single annuli.

Animals entering diapause possessed proximal-distal zones of high density cells; however, these zones did not expand with age (up to 50 days a.p.) and no mitotic figures were observed within the tissue, indicating that no mitotic activity was occurring **(** **Fig 8A** **)**. Tissue was examined from animals reared under short day conditions (12∶12 L∶D) at the following ages (hrs. a.p.): 19+/−1.4 (n = 3); 28+/−3.2 (n = 4); 43+/−3.5 (n = 4); 54+/−1 (n = 4); 68+/−1.1 (n = 3); 120+/−5 (n = 4) hrs a.p., and 50+/−2 days a.p. Tissue was fixed, treated with RNase, stained with PI and examined by MPLSM. **Fig. 8A** compares nuclear density patterns of a pre-mitotic developing animal (reared under long day conditions) with that of animals in diapause (up to 50 days); there is essentially no difference. The pre-mitotic pattern of zones of high density nuclei along the proximal and distal zones are established. However, this pattern was stable with age, and did not progress as in normal (long day) animals. Z-series analysis (not shown) revealed no mitotic figures, even during the second 24 hour period a.p. when in normal (long day) animals mitosis occurs at a low level.

Animals exiting diapause display the full temporal-spatial pattern of mitotic activity and apolysis within 36 hrs of receiving an injection of 20HE **(** **Fig. 8B,C** **)**. Twenty animals in diapause were selected from short day storage, 48–52 days a.p., chilled on ice 15–50 minutes, and injected with 20HE (20 ug per animal). After hormone injection, animals were incubated on a long day cycle (16∶8 L∶D, 27°C), and tissue was taken from each of three individuals at 0, 20, 32, 44 and 53 hrs after injection. Tissue was fixed, treated with RNase, stained with PI and examined by MPLSM. The five remaining injected animals were allowed to continue development; four of these animals were dry pharate 12 days following hormone injection, only a few hours from adult emergence, and the fifth was about 24 hrs behind, suggesting that all injected animals entered and proceeded through development relatively synchronously (staging criteria were after Schwartz and Truman [46] and Vogt *et al.* [38]. **Fig. 8B** compares tissue from an animal just injected (t_0_) with that from animals dissected at 20 and 32 hrs after hormone injection. The t_0_ tissue, approximately 50 days a.p., was essentially identical to the pre-mitotic state of a normally developing 15 hr a.p. animal **(** **Fig. 8A** **)**; no mitotic figures were observed. In contrast, active cell proliferation was observed in the 20 hr post injection tissue; mitotic figures were observed within the zone of high density nuclei and in the cell layer closest to the cuticle (**Fig. 8C** **, 20 h**) Active proliferation was also observed in the 32 hr tissue (**Fig. 8C** **, 32 h**), with the proliferative zones significantly broadened towards the mid-region of the annuli. Overall, these 20–32 hr patterns strongly resembled those observed in normal developing animals (long day) during their 48–72 hour period a.p. **(see** **Figures 4** **,** **7** **)**.

One striking difference between non-diapause and post-diapause tissue was the timing of apolysis. In non-diapause tissue, apolysis occurred consistently around 72 hrs a.p., towards the end of sensory proliferation. However, in post-diapause tissue, apolysis occurred coincident with proliferation. Of the animals exiting diapause, all showed signs of apolysis except the t_0_ animals, evidenced by patches of epithelium detached from cuticle. Two of the three 20 hr animals were entirely apolysed, two of each of the three 32 and 44 hr animals were partially apolysed and the remaining individuals were completely apolysed. All of the 53 hr animals were apolysed. This suggests that the antennal apolysis is a relatively independent process from sensory proliferation, but must be coordinated to occur about this time so that subsequent morphogenic events can follow. Apolysis does occur at similar stages in non-diapause and post-diapause animals in terms of the total progressive development of the animal, assuming diapausing animals have already undergone 36–48 hrs of development before becoming dormant.

### Ecdysteroid Regulation of Pre-apolysis Mitotic Activity

The lack of mitotic activity in animals entering diapause and the influence of ecdysteroids on diapause prompted us to investigate the influence of ecdysteroids on mitotic activity and the expansion of the mitotic zones. Rising levels of ecdysteroids are known to regulate many neuro-developmental events in *M. sexta* during the days following pupation [47]–[52]. *M. sexta* enter the pupal stage with undetectable levels of ecdysteroids. In non-diapausing pupae, ecdysteroid levels begin to rise during the initial 4 days of adult development and continue to rise to around 40% of adult development when they begin to decline until adult emergence [53], [54]. In the *M. sexta* olfactory system, rising ecdysteroids influence proliferation of glial cells associating with olfactory lobe neuropile, but not neurite outgrowth of olfactory lobe neurons [55], [56]; falling ecdysteroids stimulate expression of odorant binding proteins in the antenna [38]. To test whether the previously described mitotic activity is ecdysteroid sensitive, tissue from normally developing animals (i.e. non-diapause) was cultured for 15–24 hrs in the presence or absence of hormone, primarily using 20 hydroxy ecdysone (20HE), but also α-ecdysone (αE) and the ecdysteroid agonist RH5992 (Rohm and Hass). While 20HE is thought to be the active form of the hormone, αE appears before 20HE during adult development and may have activity during this period [54]. Relatively short culture times were chosen in part to fit in with the rapid changes in mitotic activity we had observed during this developmental period.

**Figure 9:**
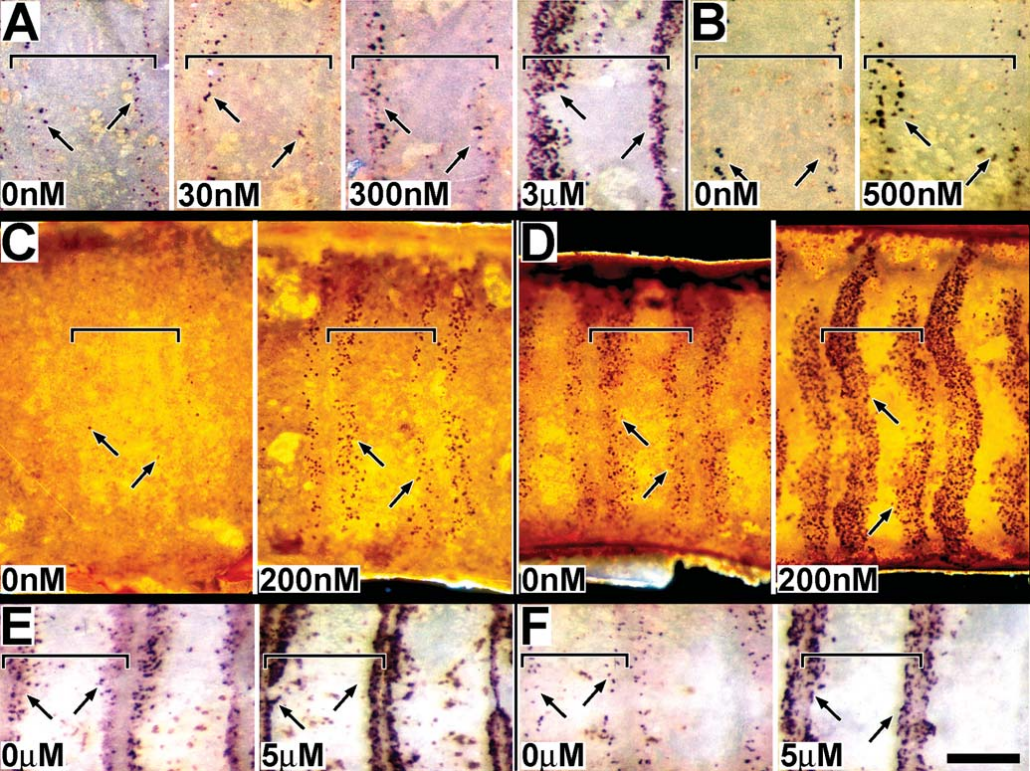
Ecdysteroid regulation of mitotic activity. A, B. Male epithelium was cultured at approximately 15 hrs a.p. (Stage 1) for 15 hrs at the indicated concentrations of 20 hydroxy ecdysone (A) or alpha ecdysone (B). BrdU was present for the entire culture period. Mitotic activity was visualized using phosphotase tagged BrdU antiserum. C, D. Male epithelium was cultured at either 33 hrs a.p. (C) or 54 hrs a.p. (D) for 15 hrs at the indicated concentrations of 20 hydroxy ecdysone. Mitotic activity was visualized using phospho-H3 antiserum. E, F. Male epithelium was cultured at approximately 30 hrs a.p. (Stage 2) for 24 hrs at the indicated concentrations of the ecdysteroid analogue RH5992. BrdU was either present for the entire culture period (E), or added after 18 hrs of culture (F). For all experiments, tissue was from normally developing animals (non-diapause). Size bar: 170 u (A,B); 234 u (C,D); 214 u (E,F).

Both 20HE and αE stimulate mitotic activity in culture. The concentration dependence of this activity is demonstrated in **Fig. 9A,B**. Tissue from individual animals was subdivided and cultured under either 4 concentrations of 20HE (0, 30 nM, 300 nM, 3 uM) or 2 concentrations of αE (0, 500 nM); six individuals selected (aged approximately 24 hrs a.p., or at Stage 1) and tested for each hormone treatment, giving equivalent results. The response to 20HE **(** **Fig. 9A** **)** showed concentration dependence, with 30 nM resembling background (0 nM) and 300 nM and 3 uM showing increasing mitotic activity. The response to 500 nM αΕ (**Fig. 9B**) was elevated above background (0 nM), and appeared similar to the response to 300 nM 20HE (**Fig. 9A**). This experiment suggested a threshold response to 20HE in culture between 30 nM and 300 nM, in the range of the threshold of 160 nM 20HE regulating cell proliferation in the developing eye of *M. sexta* [57]. The proliferative sensitivity for αE was perhaps less than for 20HE, a difference observed for proliferation in the eye [57]; in contrast with the eye, antennal response was not inhibited by higher concentrations of 20HE.

The mitotic response to 20HE was further investigated at two different developmental periods, 24–35 hrs a.p. when endogenous mitotic activity is low and 50–60 hrs a.p. when mitotic activity is high **(** **Fig. 9B,C** **)**. An upper limit of 60 hrs a.p. was chosen because older stage tissue often apolysed in culture and became lost in the culture medium. Tissue was cultured for 15 hrs+/−hormone, and mitotic activity was assayed using Phospho-H3 antibody and tissue from individual animals cultured +/−200 nM 20HE. This 20HE concentration is near the threshold determined above, and was has been suggested as the endogenous concentration at this time of development [56]. Tissue from 18 animals was tested at each stage (36 animals total) over three separate experiments, all yielding equivalent results. For the 33 hr tissue **(** **Fig. 9B** **)**, mitotic activity appeared suppressed in the absence of 20HE, but advanced in the presence of 20HE, consistent with *in vivo* tissue of appropriate age (see 46 hr tissue, **Fig. 2C**). For the 54 hr tissue (**Fig. 9C**), mitotic activity in the absence of 20HE also appeared suppressed, similar to normal tissue aged around 50 hrs, while the tissue exposed to 20HE showed an advanced state appropriate to the additional 15 hrs of culture (see **Fig. 2C**).

Preliminary to the above studies, we used the ecdysteroid hormone agonist RH5992 to investigate the viability of tissue and its mitotic response to hormonal stimulation throughout the culture period **(** **Fig. 9E,F** **)**. In the study shown, tissue was cultured +/−RH5992 (5 ug/ml) for 24 hrs, and either received BrdU for the entire 24 hr period (**Fig. 9E**), or for only the final 6 hrs of culture (**Fig. 9F**). Tissue from each of 6 individuals was subdivided and tested under 4 conditions (+/−RH5992, BrdU added at 0 or 18 hrs). Treatment with RH5992 dramatically enhanced mitotic activity as assayed by BrdU incorporation, confirming tissue viability and response through the duration of the culture period.

## Discussion

### The temporal and spatial features of pre-apolysis mitotic activity

The data presented in this manuscript focus on events occurring within the outward facing antennal epithelium (the presumptive olfactory epithelium) from the time of pupation to around the time of antennal apolysis, approximately 72 hrs. a.p. We also examined tissue before and after this time period, but only refer to data from those time periods germane to the events reported here. We did not examine the inward facing antennal epithelium which generates scale forming cells. Sanes and Hildebrand [5], [6] also described events within this outward facing epithelium, in a broader study describing overall development of the antenna. Histological sections were published showing mitotic figures within the epithelium, and tritiated thymidine birthdating was used to demonstrate that the cells comprising olfactory sensilla were generated by mitotic activities occurring during the second and third 24 hr periods following pupation. However, this earlier study did not directly examine dynamic aspects of mitotic activities during this time period; animals were injected with tritiated thymidine at various time points, and tissue was examined several days later when cellular morphologies had matured and could be identified.

In our study, mitotic activity was observed to initiate within the presumptive olfactory epithelium along the proximal and distal borders of each annulus and to expand inward into the mid-annular regions. This activity initiated within zones of high density cells which were already established as early as 2 hours after pupation. These small, high density cells were additionally distinguishable from the surrounding and larger epithelial cells based expression of *pcp*, a pupal cuticle gene; only the larger cells expressed *pcp*. Mitotic activity did not initiate until about 20–24 hrs a.p. and then only at a low level, increasing significantly around 40–48 hrs a.p. and continuing to around 72–80 hrs a.p. Additional studies by our lab suggest that this mitotic activity is complete shortly after 72 hrs a.p. (data not shown), coincident with the time of apolysis.

**Figure 10:**
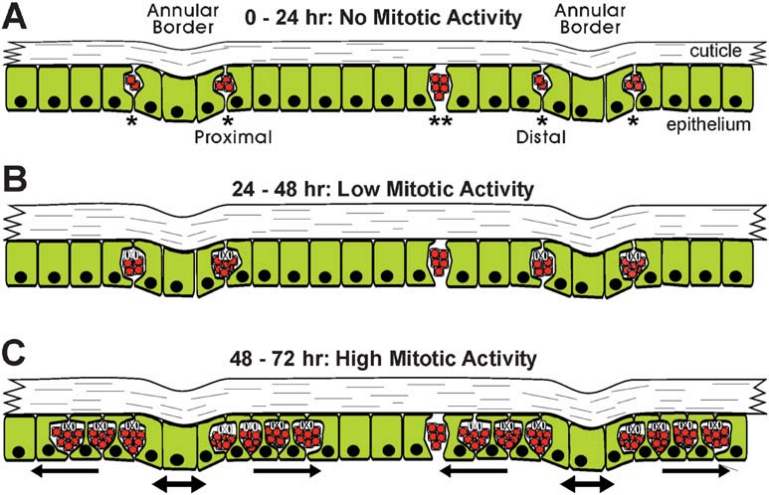
Summary of mitotic activity in olfactory epithelium from pupation to apolysis. Longitudinal cross sections of pre-apolysis male tissue are illustrated, showing pupal cuticle and underlying presumptive sensory epithelia; distal is left and proximal right. A, B and C represent the three phases of pre-apolysis mitotic activity described in the Results. High density cells are shown with red nuclei. PCP expressing cells are shown with green cytoplasm. “*” marks cells of high density zones lining distal and proximal boundaries of each annulus; “**” marks cells of mid-annular high density clusters. High density zones and mid-annular clusters are present at pupation; high density zones expand in space and cell number with the onset of mitotic activity, but mid-annular clusters remain constant in cell number throughout this period. See text.

We have summarized these events in **Figure 10** **,** dividing the time from pupation to apolysis into three approximately 24 hr long periods. During the first 24 hr period **(** **Fig. 10A** **)**, no mitotic activity was observed. Inter- and intra-annular epithelial regions are distinguishable by cell shape and by the morphology of the overlying cuticle; olfactory sensilla develop within the intra-annular regions. High density zones of small cells line the proximal and distal borders of each annulus within the presumptive sensory epithelium, observable as early as 2 hrs a.p.; the distal zone is consistently broader than the proximal zone and is discontinuous at the longitudinal mid-line (**see** **Fig. 3A**). Cells expressing and secreting pupal cuticle genes (e.g. *pcp*) fill the remainder of the epithelium, including the mid and inter-annular regions; large *pcp* expressing cells are also present within the high-density zones where they intermingle with the smaller cells (which do not express *pcp*). In addition, small clusters of high density nuclei (cells) are present within the mid-annular region; these small cells do not express *pcp*, and their clusters are not disrupted by epithelial cells expressing *pcp*.

Mitotic activity initiates at the onset of the second 24 hr period a.p. **(** **Fig. 10B** **)** but at a low rate compared to the third 24 hr period a.p. (**Fig. 10C**). During these later periods, the proximal and distal zones broaden towards the mid-annular region, meeting around the time of apolysis (72–80 hrs a.p.). Towards the end of the third 24 hr period, the inter-annular regions were observed to broaden as well, possibly through changes in cell shape; no mitotic activity was observed in the inter-annular regions up to 72 hrs a.p. Mitotic divisions occurred within the nuclear layer closest to the cuticle, with cells accumulating away from the cuticle, in the direction of the hemolymph. Expression of *pcp* occurred in cells distributed throughout the epithelium, but not within the small cells of the high density zones. Within the proximal and distal zones, the nuclei of cells expressing *pcp* were observed basal to the nuclei of high density cells; *pcp* expressing cells spanned the epithelium to the cuticle, intermingling with the high density cells.

We suggest that the peripheral high density zones give rise to trichoid and basiconic sensilla, while the mid-annular clusters give rise to coeloconic sensilla. Such distinctions would be consistent with the distribution of the numbers and distributions of these sensilla types in the adult male *M. sexta* antenna [8], with the studies of Sanes and Hildebrand [6] which linked the observed mitotic activities to the formation of trichoid and basiconic sensilla, and with findings in *D. melanogaster* that these three classes of sensilla are differentially regulated by distinct proneural genes at different times of development [28], [29].

The initial spatial pattern of the proximal/distal high density zones and mitotic activity is similar to the distribution of trichoid sensilla in the adult male *M. sexta* antenna: both are distributed along the proximal/distal annular borders and both are discontinuous along the mid-annular axis of the distal zones. This suggests that trichoid sensilla might originate from cells of the peripheral regions and that basiconic sensilla might originate from cells of the mid-annular regions that result from mitotic expansion, and that the formation of trichoid sensilla might thus precede that of basiconic sensilla. Such a timing difference would be consistent with findings that sensory nerve axons of trichoid sensilla enter the brain prior to those of basiconic sensilla [31]. If it is true that the male trichoid sensilla emerge from the initial peripheral zones, then it is curious that mitotic activity in female antennae also initiates within proximal-distal zones and expands inward **(** **Fig 2B** **)**, as female antennae lack the long trichoid sensilla of male antennae. However, female antennae do have a different distribution of sensilla in the peripheral vs. mid-annular regions [9], and the spatial pattern of female mitotic activity may reflect this difference.

### Temporal asymmetry of mitotic activity: ecdysteroids and diapause

**Figure 11:**
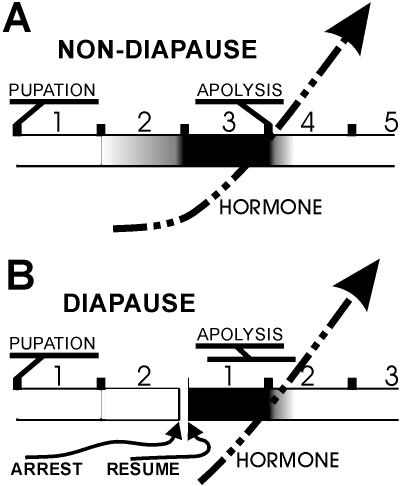
Influence of ecdysteroids and diapause on the onset of mitotic activity. Horizontal time line is marked in days a.p. (A,B) or days after development resumes (B). Shading in time line represents mitotic activity, with black the highest level of activity. Dashed arrow represents increasing levels of circulating ecdysteroids. See text for details.

Tissue culture experiments demonstrate that mitotic activity and expansion are sensitive to ecdysteroid levels (**Fig. 11**). Mitotic activity also appears to be sensitive to diapause **(** **Fig. 8** **)**, which is also sensitive to ecdysteroids. We suggest that the change in mitotic rates around 24 and 48 hrs a.p. in non-diapausing animals reflects both the hormonal states of the animal at these times and a developmental accommodation to diapause (**Fig. 11**).

In both non-diapausing and diapausing animals, the presumptive sensory epithelium initiates development, after pupation, with two zones of high density cells lining the proximal and distal borders of each annulus and a large field of low density cells filling the middle annular regions. Development is continuous for non-diapausing animals, but arrests at around 48 hrs a.p. in diapausing animals. In non-diapausing animals, mitotic activity initiates within the high density zones beginning around 20–24 hrs a.p. and dramatically increases beginning around 40–48 hrs a.p. with the subsequent expansion of the high density zones into the mid-annular region. In animals entering diapause, no mitotic activity was observed during this period; the high density zones remained a consistent size once the epithelium stabilized following pupation and for the duration of diapause. However, once diapause was terminated and development re-initiated, mitotic activity quickly appeared within the high density zones and the zones rapidly expanded toward the mid-annular region within 36 hrs (**Fig. 8**) In addition, for non-diapausing animals, mitotic activity is sensitive to ecdysteroids in a dose dependent manner (**Fig. 9**).

Bowen and colleagues [40], [41] showed that developmental arrest in diapausing animals at around 48 hrs a.p. is due to a lack of ecdysteroid production. We suggest that in non-diapausing tissue, the epithelium is primed to initiate proliferation, and does so to a small extent during the second 24 hr period a.p., perhaps in response to very low levels of ecdysteroid present at that time. However, to accommodate a developmental program following diapause, the tissue is organized to perform the bulk of its proliferative activity very rapidly, within a 24–36 hr period. This period is approximately the third 24 hr period a.p. in non-diapausing animals, but in diapausing animals is the initial 24–36 hr period following the reinitiation of development. In this context, the low level activity observed during the second 24 hr period a.p. in non-diapausing animals may represent a leakage activity that precedes the full blown proliferative activity that initiates around 48 hrs a.p. in response to a more robust release of hormone. It seems unlikely that diapause-related developmental arrest is solely controlled by the presence or absence of ecdysteroids; antennal tissue entering diapause may lack capability to respond to hormone through lack of expression of hormone receptors.

### Comparisons to other systems

Sensitivity to diapause has also been proposed for eye development in *M. sexta. A* dorsal-ventral furrow forms in the eye imaginal primordium about 2 days before *M. sexta* pupation; mitotic activity occurs along the anterior and posterior borders of this furrow which migrates across the presumptive retinal epithelium over the course of 6 days, until about day 4 a.p. [58]–[60]. In diapausing animals, furrow migration arrests about 48 hrs a.p., with the furrow progressed only about 1/3 of the way [57], [61]. Furthermore, in culture, mitotic activity associated with the furrow could be reversibly started and stopped by adjusting levels of ecdysteroid, suggesting that ecdysteroids are, in part, regulating developmental arrest of the eye for animals entering diapause [57].

The mitotic activities that directly produce sensory cells in the *M. sexta* eye initiate during the larval stage, soon after feeding has halted but about 2 days before the larval-pupal molt [57], [58]. The equivalent activities in the antenna do not initiate until at least 24 hrs after the larval-pupal molt, at least for the majority of olfactory cells ([6], and this study). One exception to this in the antenna is the clusters of cells observed in the mid-annular region shortly after the molt. These have a distribution similar to that observed for coeloconic sensilla in adult male antenna [8], and may thus represent this sensilla class. Presumably the proliferative events that gave rise to these small cell clusters occurred much earlier, at least 12 hrs before pupation, as we observed no mitotic activity from 12 hrs before pupation (data not shown) to 20–24 hrs after pupation based on BrdU injection studies. Another possible exception is the zones of high density cells lining the proximal-distal borders of each annulus; these cells were observed as early as 2 hrs a.p., and if these cells derived from mitotic events, those events must also have occurred earlier than 12 hrs before pupation. Alternatively, the high density zones may have become organized by recruitment rather than by mitosis, in a manner similar to the recruitment of sensory progenitor cells in the developing olfactory epithelium of *D. melanogaster* [28], [29].

In *D. melanogaster*, two proneural genes, *atonal* and *amos* regulate the development of coeloconic sensilla (*atonal*) vs. basiconic and trichoid sensilla (*amos*) [26], [28], [29], [62], [63]. Expression of *atonal* and *amos* occurs in non-overlapping zones within the presumptive olfactory epithelium; sensory precursor cells giving rise to coeloconic sensilla emerge from within the *atonal* zones, while those giving rise to basiconic and trichoid sensilla emerge from within the *amos* zones [28]. The *atonal* dependent sensory precursors (coeloconic) appear before *amos* dependent sensory precursors (basiconic and trichoid); *amos* expression and the production of *amos* dependent sensory precursor cells (basiconic and trichoid) persists well into the pupal stage, and these zones expand to fill the entire olfactory epithelium [28]. These observations seem parallel to events we have observed in *M. sexta*. The developing presumptive olfactory epithelium of *M. sexta* is also divided into non-overlapping zones, a mid-annular zone containing a small number of cell clusters we speculate will form coeloconic sensilla and two peripheral zones of high density cells we speculate will generate basiconic and trichoid sensilla. Pre-pupation mitotic activity presumably generates the mid-annular coeloconic cell clusters, while post-pupation mitotic activity generates basiconic and trichoid cells. Similar to expansion of the *amos* expression zones in *D. melanogaster*, mitotic activity initiating in the peripheral zones of *M. sexta* expands to fill the entire olfactory epithelium. We would like to understand the extent to which olfactory development in these two systems, *Drosophila* and *Manduca*, have diverged. To that end, we are currently characterizing genetic markers, especially transcriptional regulators, common to both fly and moth that differentiate the patterns and cell types described in this study.
